# Clavicular cortical hyperostosis: new autoinflammatory entity or part of the juvenile spondyloarthropathies clinical picture?

**DOI:** 10.1186/1546-0096-9-S1-P299

**Published:** 2011-09-14

**Authors:** L Lamot, LT Bukovac, F Borovecki, K Vlahovicek, S Sumic, K Gotovac, F Bingula, M Harjacek

**Affiliations:** 1 Children’s Hospital Srebrnjak, Division of Rheumatology; 2 Department of Pediatrics, University of Zagreb School of Medicine, Zagreb, Croatia; 3 Department for the functional genomics, Center for translational and clinical research, University of Zagreb School of Medicine, Zagreb, Croatia; 4 Bioinformatics Group, Department of Molecular Biology, Faculty of Science, University of Zagreb, Zagreb, Croatia

## Background

Clavicular cortical hyperostosis (CCH–variant of chronic recurrent multifocal osteomyelitis) is characterized by unilateral sterno-clavicular swelling. In adults, it is associated with spondyloarthropathies, but the possible association to juvenile spondyloarthropathies (jSpA) is currently unknown.

## Aim

To identify genes with disease-specific expression patterns of patients diagnosed with CCH and healthy controls, and compared it to the jSpA patients.

## Patients and methods

Peripheral blood samples of 5 new-onset, untreated patients with CCH, 11 new-onset, untreated jSpA patients and 4 healthy controls were analyzed for expression patterns using Human Genome U133 PLUS 2.0 GeneChip, Affymetrix, (54675 probes). Processing of raw signal intensities and annotation of probesets was preformed. The RT-PCR was used to confirm differential gene expression in total of 9 CCH, 11 healthy controls and 32 jSpA patients.

## Results

Complex statistical analysis of gene expression patterns in patients with CCH identified 974 differentially expressed genes at statistical cutoffs fold change 1.5 (p<0.05, max>100). Genes differentially expressed in patients with CCH were compared to the expression profiles of jSpA patients, and the analysis showed overlap in 282 genes.

## Conclusions

CCH patients exhibit complex patterns of expression in genes related to inflammatory response (STAT3 downregulation), B-cell activation, MAP kinase (TRPM3 upregulation) chromatin modulation and transcription, cell death and apoptosis, and most interestingly, genes closely linked to autoinflammatory diseases (PTPN12 and MEFV). Additionally, profiles of patients with CCH and jSpA showed significant concordance in expression of genes linked to autoinflammatory (TLR-4, PTPN12) and autoimmune diseases (STAT3, CD36).

